# A chip-less and battery-less subharmonic tag for wireless sensing with parametrically enhanced sensitivity and dynamic range

**DOI:** 10.1038/s41598-021-82894-x

**Published:** 2021-02-12

**Authors:** Hussein M. E. Hussein, Matteo Rinaldi, Marvin Onabajo, Cristian Cassella

**Affiliations:** Electrical and Computer Engineering Department, Northeastern University, Boston, USA

**Keywords:** Electrical and electronic engineering, Electronic and spintronic devices, Characterization and analytical techniques, Design, synthesis and processing, Nonlinear phenomena

## Abstract

Massive deployments of wireless sensor nodes (WSNs) that continuously detect physical, biological or chemical parameters are needed to truly benefit from the unprecedented possibilities opened by the Internet-of-Things (IoT). Just recently, new sensors with higher sensitivities have been demonstrated by leveraging advanced on-chip designs and microfabrication processes. Yet, WSNs using such sensors require energy to transmit the sensed information. Consequently, they either contain batteries that need to be periodically replaced or energy harvesting circuits whose low efficiencies prevent a frequent and continuous sensing and impact the maximum range of communication. Here, we report a new chip-less and battery-less tag-based WSN that fundamentally breaks any previous paradigm. This WSN, formed by off-the-shelf lumped components on a printed substrate, can sense and transmit information without any need of supplied or harvested DC power, while enabling full-duplex transceiver designs for interrogating nodes rendering them immune to their own self-interference. Also, even though the reported WSN does not require any advanced and expensive manufacturing, its unique parametric dynamical behavior enables extraordinary sensitivities and dynamic ranges that can even surpass those achieved by on-chip sensors. The operation and performance of the first implementation of this new WSN are reported. This device operates in the Ultra-High-Frequency range and is capable to passively and continuously detect temperature changes remotely from an interrogating node.

In the last decades, the continuously expanding Internet-of-Things (IoT) has created a plethora of new exciting possibilities within recently developed smart applications for structural health monitoring^1^, environmental surveys^2^, smart logistics^3^ and more. Nevertheless, such possibilities could be fully exploited only if low-cost and higher sensitivity wireless sensor nodes (WSNs) able to operate uninterruptedly were available for a massive-scale deployment^4–7^. For instance, the ability to strategically distribute thousands of such desired WSNs would aid to promptly detect and localize any behavioral anomalies in the structures of buildings, bridges and more, hence permitting to monitor their structural integrity^8^. Similarly, the same ability would allow to timely identify the occurrence of a fire^9–12^ in both indoor and outdoor settings, thus improving the safety of individuals and greatly reducing the losses in agriculture and in national resources that are more and more often experienced today. Also, according to recent studies, more than 20% of the food produced every year in the sole United States is wasted due to items in cold manufacturing and delivery chains^13^ exposed to not suitable temperatures, causing a financial loss of more than 200 billion dollars. The inadequate refrigeration of perishable food is also responsible for serious food-borne illnesses that annually cause more than one hundred thousand hospitalizations and thousands of deaths just in the US^13^. By enabling low-cost WSNs with long enough lifetime to continuously monitor all the processes in cold-chains and to promptly and reliably identify any specific items exposed to inadequate temperature, it would be possible to significantly lower these dramatic numbers, hence mitigating their serious consequences.

**Figure 1:**
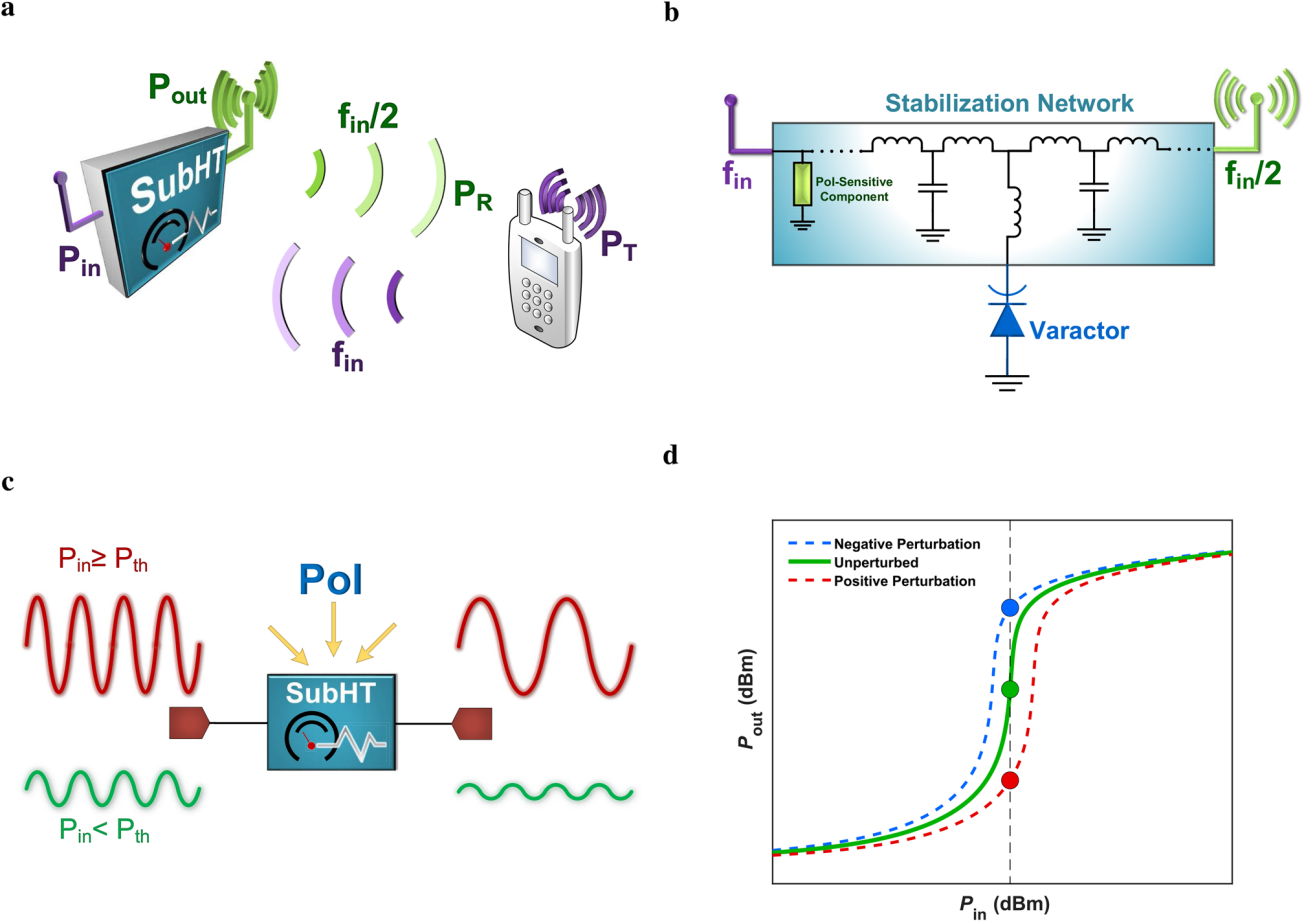
A Sub-Harmonic Tag (SubHT) and its unique operational features. (**a**) Schematic of an envisioned SubHT-enabled wireless sensing architecture. It allows to passively and remotely sense any targeted *PoI*s. Also, the sensed information is radiated back from the SubHT towards the interrogating node by using a passively generated carrier frequency ($f_{in}$/2) that is half of the interrogating frequency ($f_{in}$). (**b**) Circuit schematic representation of a generic SubHT. This includes a varactor and a passive network of off-the-shelf lumped components acting as a stabilization network for the large-signal periodic regimes driven by the portion ($P_{in}$) of the power transmitted by the interrogation node ($P_{T}$) that is received by the SubHT. Also, this network embodies one component that is sensitive to the specific *PoI* and that is responsible for the activation of the unique dynamics leveraged by the SubHT to sense the *PoI*. (**c**) A graphic representation of the typical input and output signals characteristics of a SubHT, for input power levels ($P_{in}$) lower (in green) or higher (in red) than the SubHT parametric power threshold ($P_{th}$). (**d**) Typical output power ($P_{out}$) versus $P_{in}$ characteristic of a SubHT when not perturbed (in green) by the *PoI* or, alternatively, when subject to a positive (in red) or negative (in blue) variations of the *PoI*.

Any existing WSN^14,15^ used for remote sensing applications can be seen as the combination of a sensing system and a radio frequency (RF) front-end responsible to transmit and receive electromagnetic signals. The sensing system relies on a sensor to detect the variations of a specific parameter-of-interest (*PoI*) with a sensitivity that strongly depends on the adopted sensing technology. In particular, the development of advanced manufacturing processes has recently enabled sensitive on-chip micro- and nano-electromechanical (MEM/NEM) physical^12,16^ and chemical^17,18^ sensors, consuming near-zero stand-by powers. Yet, the majority of the existing WSNs, including those using such miniaturized new sensors, still require considerable amounts of energy to transmit the sensed information to any other interrogating nodes or readers within the same network. As a result, they must rely on on-board batteries or, alternatively, on integrated harvesting circuits^19^, scavenging energy from the environment and use it to temporally sustain the transmission capabilities. Thanks to the recent advancement in zero-power sensor technologies^12^, battery-powered WSNs can nowadays achieve extremely long lifetimes (nearly 10 years, limited by the self-discharge of their batteries) when deployed to detect time-critical but relatively rare events (i.e. operating predominantly in off- but alert-mode). Nevertheless, such lifetimes can be abruptly reduced to just few months when, instead, WSNs need to sense and transmit information many times per hour, thus demanding orders of magnitude higher average power levels than what consumed during their stand-by operational mode. In such more elaborate operational scenario, frequent periodical battery replacements are required, hence leading to high maintenance costs that can even be unsustainable when WSNs are deployed at hardly reachable locations or in harsh environments. Also, the increase of the number of deployed battery-powered WSNs is generating a fast growing environmental concern regarding the disposal of batteries in landfills. Similarly, any WSNs relying on on-chip harvesting circuits are also hardly usable when a frequent detection of any *PoI*s is required. In fact, both the maximum communication range and the highest detection rate achievable through these WSNs are severely affected by the inability of the currently available rectifying circuits to exhibit acceptable efficiencies when receiving RF power levels significantly lower than 1 mW^20^. So, in order to enable WSNs that can be frequently interrogated without relying on any batteries or harvesting circuits, a growing attention has been recently paid to chip-less and battery-less tag-based WSNs. These WSNs are printable on easily disposable substrates, thus enabling exceptionally low manufacturing costs^7^, while being equipped with sensing capabilities. Yet, in order to achieve a small size and a long communication range,while rendering any interrogating nodes able to separate the transmitted and received data streams, the existing chip-less and battery-less tag-based WSNs must rely on advanced resonant components with exceptionally high quality factors (*Q*), like surface acoustic wave (SAW)^21^ devices. Although the use of such high-*Q* components comes with significantly higher manufacturing costs^7^, these devices are key, when used in conventional tag-based WSNs, to ensure that any interrogating nodes can distinguish the received sensed information from their self-interference and from any occurring environmental electromagnetic echos of their output signals. Just recently, in order to avoid using any expensive high-*Q* components, a class of chip-less and battery-less tag-based WSNs known as *harmonic* *tags* (HTs) has been proposed^22,23^. These WSNs rely on unbiased nonlinear devices, such as varactors or Shottkey diodes, to deliver the sensed information through output signals that have twice the frequency of the interrogating ones, thus being easily distinguishable, once received by the interrogating nodes, from any undesired signals with the same frequency used by the interrogating one. Yet, the output signals of harmonic tags show power levels that are lower than those of their input signals by a large amount known as conversion loss (CL) that, depending on the technology of the nonlinear variable capacitor, can even exceed 35 dB^23^ when the received input power levels are lower than − 15 dBm. In addition, since the sensed information is transmitted at twice the frequency of the interrogating signals, harmonic tags inherently suffer from a 6 dB higher path-loss than traditional single-frequency counterparts, thus further reducing the signal-to-noise ratio (SNR) at the receiver of their interrogating nodes and, consequently, the maximum communication range.

In this Article, we present the first prototype of a novel class of chip-less and battery-less tag-based WSNs, referred to as *sub*-*harmonic* *tags* (SubHTs) (Fig. 1a). SubHTs break any previous paradigm related to the design of chip-less and battery-less tag-based WSNs by making it possible to remotely and continuously sense *PoI*s with extraordinary sensitivities and dynamic ranges, yet relying on low-cost off-the-shelf lumped components assembled on printed substrates. Also, similarly to any previously reported harmonic tags, SubHTs transmit the sensed information over a dedicated channel, distant from the one leveraged to interrogate them. However, differently from any previous counterparts, this full-duplex characteristic is achieved by strategically operating in regions where a parametrically originated *period*-*doubling* mechanism is active. This mechanism allows SubHTs to transmit the sensed information through a dedicated passively generated carrier frequency ($f_{out}$), which is half of the one used by the interrogating signal ($f_{in}$ = $2f_{out}$). Regardless of the very low input power levels ($<-18$ dBm) at which SubHTs can operate and despite the fact that no DC biasing voltage is used, SubHTs generate the output signal from the received input power more efficiently than harmonic tags, thus enabling significantly lower CL-values. Furthermore, since for any chosen $f_{in}$ value $f_{out}$ is always one fourth of the output frequency that would be used if harmonic tags were adopted, SubHTs inherently enable a 12dB reduction in the path-loss affecting the portion of their output signal reaching the interrogating nodes. These unique features enable orders of magnitude higher SNRs at the receiver of the interrogating nodes than what has ever been possible to achieve through harmonic tags, hence paving the way towards a more accurate wireless sensing and a longer communication range. Furthermore, we show that the unexplored parametric dynamics leveraged by SubHTs^24–27^ also allow to massively boost the sensitivity and the dynamic range attained by off-the-shelf commercial sensors, thus providing the means to achieve superior sensing capabilities without requiring advanced on-chip sensors like the recently developed MEM/NEM components.

In order to demonstrate the unique characteristics exhibited by SubHTs, the operation and performance of the first SubHT prototype made of off-the-shelf lumped components are described here. This device operates at $f_{in}$ equal to 886 MHz and remotely measures temperature at 4 m from a complementary interrogating node. Despite the fact that this SubHT is not relying on any advanced components with high temperature-sensitivity and high dynamic range but only on a commercial off-the-shelf thermistor, it can show a sensitivity ($S_{max}$) and a dynamic range that are orders of magnitude higher than what is achievable when the same thermistor is used as a separate sensor. In the next section, we will discuss the general principle of operation of SubHTs. Afterwards, we will focus on the main design and performance characteristics of the built SubHT prototype.

## Principle of operation

Independently of the targeted sensing parameter, any SubHT can be described as a two-port electrical network formed by an un-biased variable capacitor and a set of lumped electrical passive elements (Fig. 1b). This set includes a component, such as a separate commercial off-the-shelf sensor, with an electrical impedance dependent on the specific *PoI*. The two ports of any SubHTs are connected to properly sized antennas, enabling the simultaneous reception and transmission of signals from and to the interrogating nodes. The technology (planar, wire, aperture, etc.) and design characteristics of such antennas can be chosen based on the targeted application and other system level requirements. Depending on the strength and on the frequency of its input signal, a SubHT can exhibit operational regions where it undergoes a period-doubling mechanism^26,28^. In such regions, it relies on the energy coming from the interrogating node, at a frequency $f_{in}$, to passively generate a strong output signal at $f_{in}$/2 (i.e. $f_{out}$), which is radiated back to the interrogating node. The activation of such period-doubling mechanism (Fig. 1c) occurs through a super-critical bifurcation^28^ triggered by the power ($P_{in}$) of the SubHT input signal. In particular, for $P_{in}$ values exceeding a certain threshold (known as parametric threshold, $P_{th}$), SubHTs exhibit a steep but continuous $P_{out}$ versus $P_{in}$ characteristic (Fig. 1d), where $P_{out}$ is the output power at $f_{out}$ delivered to the antenna used for transmission. $P_{th}$, which designates the minimum input power at which a SubHT can operate, is set by the junction capacitance and tuning range exhibited by the adopted varactor, along with the impedances that such variable capacitor sees at both $f_{in}$ and $f_{out}$^27^. These impedances are set by the equivalent passive network formed by the selected lumped components excluding the varactor. Such network acts as a *stabilization network* for the non-autonomous periodic regimes generated by the interrogating signal through the large modulation of the varactor’s capacitance. The SubHT lumped components are selected to minimize $P_{th}$ given a desired $f_{in}$ value. Yet, by including the chosen component with impedance dependent on the targeted *PoI* within the stabilization network, any change in the strength exerted on the SubHT by such *PoI* results into a corresponding change of $P_{th}$, hence activating a previously unexplored dynamical behavior that is leveraged here for the first time (Fig. 1d). Due to the steep slope of the $P_{out}$ versus $P_{in}$ characteristic for $P_{in}$ approaching $P_{th}$ and due to the fact that the power at $f_{out}$ is only generated for $P_{in}$ higher than $P_{th}$ and no period-doubling mechanism is active for $P_{in}$ lower than $P_{th}$ (Fig. 1c), any induced variations of $P_{th}$ produces an extremely large change of $P_{out}$ and, consequently, of the power received ($P_{R}$) at $f_{out}$ by the interrogating device. Such change can span over several orders of magnitude, even when only small perturbations to the SubHT operational point are caused by the *PoI*. This unique dynamical feature provides the means to achieve a sensitivity to the *PoI* and a dynamic range that greatly exceed what is possible when the selected SubHT component with electrical response dependent on the *PoI* is used as an independent sensor. In other words, SubHTs pave the way towards tag-based WSNs that can surpass, electronically, the limited sensitivity of their sensitive element, instead of requiring more advanced technologies that demand higher fabrication complexities or special operating conditions unsuitable for a massive-scale deployment. By analyzing the power received ($P_{R}$) at $f_{out}$, the interrogating node can then remotely assess the strength of the *PoI* at the SubHT location. Thanks to the fact that SubHTs can couple the sensed information to a different carrier frequency from the one used to interrogate them, they don’t need high quality factor components. Instead, SubHTs enable full-duplex transceiver architectures for the interrogating nodes, simply relying on two filtering components, centered at $f_{in}$ and $f_{out}$, to separate simultaneously transmitted and received data streams.

**Figure 2:**
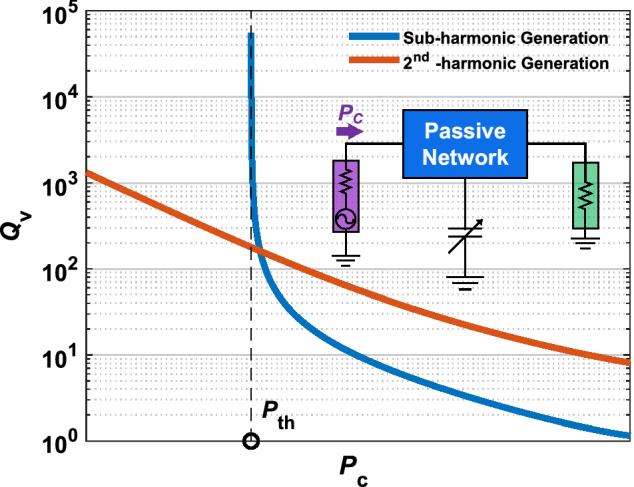
The power dependent quality factor exhibited by an ideal largely modulated reactance used for frequency division or frequency doubling. Typical trends of $Q_v$ versus $P_c$ attained through circuit simulations and relative to an ideal nonlinear reactance, independently used by two same-order and same-topology passive circuits respectively optimized for frequency division by two (in blue) or for frequency doubling (in red). More details about the simulation strategy we followed to extract these trends are reported in Supplementary Fig. 4.

Furthermore, it is also crucial to point out that due to the SubHTs unique dynamics, the generation of their sub-harmonic output signal from $P_{in}$ can be significantly more efficient than the corresponding production of a high-order harmonic in any harmonic tags. Such unexplored feature is enabled by the capability of any parametric systems operating above threshold to more efficiently transform the energy stored by their nonlinear reactances at the driving frequency into power at the desired sub-harmonic output frequency. This can be verified, for instance, by monitoring the different trends of the power dependent quality factor ($Q_{v}$, Supplementary Fig. 4) exhibited by an ideal lossless nonlinear reactance, connected either to a lossless stabilization network to enable a frequency division with minimum $P_{th}$ or to a circuit exploiting the same topology used for the stabilization network, yet engineered to allow a frequency doubling functionality with minimum CL. These frequency dividing and frequency doubling systems operate with the same input frequency ($f_c$) and input power ($P_c$) but with an output frequency being either half or twice $f_c$ (Fig. 2). As we rely on these two exemplificative systems to assess the capability of the same nonlinear reactance to generate different desired output frequencies, $Q_{v}$ is of great interest as it maps the ratio between the imaginary part of the modulated reactance impedance at $f_c$ and its nonlinearly generated resistance ($R_{conv}$). This resistance captures the effects of the capacitance modulation at $f_c$ on the energy transformation between input and output frequencies, thus progressively increasing as higher $P_c$ values are used. Also, differently from CL, $Q_v$ is independent of the matching characteristics relative to the circuits ports, hence being a more adequate parameter to assess the intrinsic conversion capabilities granted by the same nonlinear reactance when used in the two analyzed systems. In particular, while $Q_{v}$ diverges for $P_c$ tending to zero (for the frequency doubling circuit) or to $P_{th}$ (for the parametric frequency dividing circuit), due to the decreasing capacitance modulation lowering $R_{conv}$, it progressively reduces as $P_c$ is increased. In particular, by comparing the trends of $Q_v$ versus $P_c$ relative to the two investigated circuits, a significantly lower $Q_v$ value can be found, for $P_c$ higher than $P_{th}$, when the nonlinear reactance is used to parametrically generate a sub-harmonic output signal, like in SubHTs, rather than create a second harmonic one, like in any previously reported harmonic tags (Fig. 2). As a result, for low $P_c$ values, the CL value achieved by SubHTs can be smaller than the corresponding value in harmonic tags, thus allowing to increase the SNR at the receiver of the interrogating nodes without requiring more power to be transmitted by the same nodes. Moreover, it is important to point out that since $P_{out}$ has a frequency that is one fourth of the output one adopted for the same driving frequency by harmonic tags, the SNR improvement enabled by SubHTs is further amplified (by 12dB in free-space) due to a reduction in the path-loss affecting $P_{out}$ before reaching any interrogating nodes.

**Figure 3:**
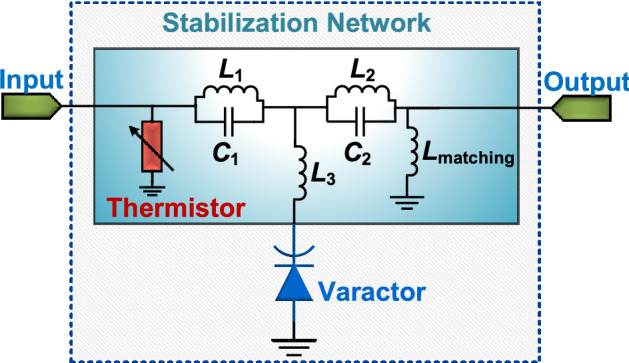
Circuit schematic of the realized SubHT for temperature sensing. The components forming the stabilization network of the built SubHT are shown, including the off-the-shelf thermistor used to activate the unique temperature-sensitive dynamics leveraged during the sensing operation. The components values for the reported prototype are: $L_1 = 39$ nH, $L_2 = 22$ nH, $L_3 = 22$ nH, $L_{matching} = 1.8$ nH, $C_1 = 1.5$ pF, $C_2 = 1.4$ pF, the thermistor’s resistance at ambient temperature is 2.6 $\Omega$ and the varactor’s rest capacitance is 1.24 pF.

## An Ultra-High-Frequency (UHF) SubHT for temperature sensing

**Figure 4:**
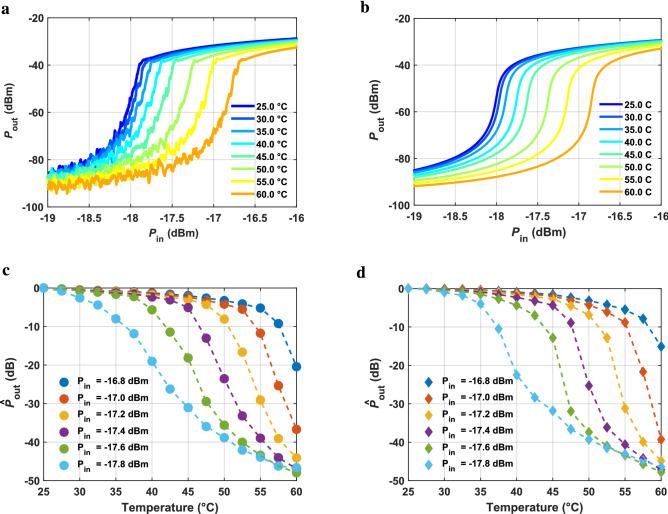
Evaluation of the sensing capabilities of the fabricated SubHT. (**a, b**) Measured (**a**) and simulated (**b**) $P_{out}$ versus $P_{in}$ trends of the fabricated SubHT, at $f_{in}$ = 886 MHz and for different temperatures (*T*s) ranging from 25 $^\circ$C to 60 $^\circ$C. (**c, d**) Measured (**c**) and simulated (**d**) $P_{out}$ versus *T* trends of the fabricated SubHT for different $P_{in}$ values close to the $P_{th}$ value extracted at 25 $^\circ$C. All the reported curves (**a**–**d**) were extracted through a wired characterization experiment.

**Figure 5:**
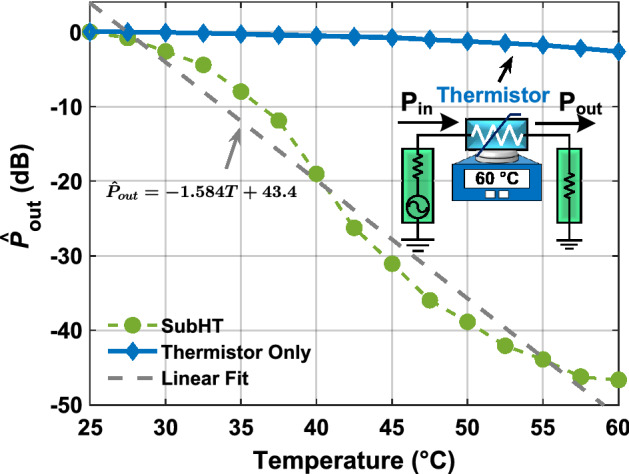
Surpassing the limits in the achievable sensitivity. A comparison of the ${\hat{P}}_{out}$values attained by the built SubHT (in green) at $P_{in} = -17.8$ dBm, for the different investigated temperatures, with the corresponding ${\hat{P}}_{out}$values (in blue) that would be attained, instead, if the thermistor used by the SubHT was individually utilized as the temperature sensor. For clarity, the circuit schematic used for the evaluation of the latter case is also displayed in the in-set. Also, a linear fitting line for ${\hat{P}}_{out}$ versus *T* is shown (in gray) together with its corresponding equation.

In order to experimentally demonstrate the unique performance features of SubHTs, we decided to build a SubHT prototype targeting a remote and continuous temperature (*T*) sensing. This prototype was designed and assembled on a printed circuit board (PCB) made of FR-4, relying on off-the-shelf lumped components including two capacitors ($C_{1}$ and $C_2$), four inductors ($L_1$, $L_2$, $L_3$, $L_{matching}$), one varactor and a commercial thermistor (Fig. 3). The values and model-numbers of all components in the circuit, the board layout description and a picture of the fabricated SubHT are available in the Supplementary Material (Supplementary Fig. 1 and Supplementary Table 1). The thermistor was used as the required sensitive element in the SubHT stabilization network, allowing its unique temperature-sensitive dynamics. Following our recent theoretical investigation on the stability of varactor-based parametric systems^27^, and given the impedance exhibited at room temperature by the selected thermistor, the inductors and capacitors of the built SubHT were selected to minimize $P_{th}$ at $f_{in}$ equal to 886 MHz. This was done by satisfying four resonant conditions allowing the maximum voltage level across the varactor at $f_{in}$, the minimum leakage of $P_{out}$ towards the receiving antenna and the lowest impedance magnitude seen by the varactor at $f_{out}$. In other words, such design conditions simultaneously enable the largest modulation depth of the varactor’s capacitance, the highest output power, and the lowest loss that is to be parametrically compensated in order to trigger the desired sub-harmonic oscillation in the circuit. We characterized the unique temperature sensing capabilities of the SubHT by placing it on a digitally controlled hotplate to vary the *T* value at the SubHT location from 25 $^\circ$C to 60 $^\circ$C with a step of 2. 5$^\circ$C. The SubHT input and output ports were connected to two synchronized network analyzers, respectively acting as a 50$\Omega$ signal generator at $f_{in}$ and as a 50 $\Omega$ power meter at $f_{out}$. The measured $P_{out}$ versus $P_{in}$ characteristics for all the explored *T* values are reported (Fig. 4a), along with the closely matching corresponding ones we found through circuit simulations (Fig. 4b). As expected, a super-critical bifurcation was found for all the explored *T* values, marking the transition between the SubHT operational regions without frequency division and the ones with frequency division. In particular, $P_{th}$ values as low as $-$ 18.5 dBm were measured along with CL values approaching 21 dB, which are significantly lower than the ones of any reported harmonic tags relying on unbiased nonlinear reactances^23^ to avoid using batteries or energy harvesters. Furthermore, as the temperature at the SubHT location was varied, we noticed a clear monotonic increase of $P_{th}$ caused by a temperature-driven change of the impedance seen by the varactor at $f_{in}$. Due to the steep slope of the $P_{out}$ versus $P_{in}$ characteristic exhibited in proximity of the super-critical bifurcation, such a shift in $P_{th}$ can produce a large variation of $P_{out}$ that provides the means to achieve the superior sensing capabilities reported in this work. In fact, by strategically selecting a $P_{in}$ value close to the specific $P_{th}$ value measured at 25 $^\circ$C, this SubHT can obtain extraordinary sensitivities and dynamic ranges that cannot be reached otherwise. This was confirmed, through both direct measurements and circuit simulations, by extracting the corresponding $P_{out}$ values for different $P_{in}$ close to $-$ 18.5 dBm and when considering the same analyzed *T* values. The extracted values (${\hat{P}}_{out}$) from both our measurements and simulations, normalized to the corresponding $P_{out}$ values at 25 $^\circ$C, are shown in Fig. 4c and Fig. 4d respectively. As evident, the built SubHT can exhibit remarkable ratios ($\Delta$ ${\hat{P}}_{out}$) between the ${\hat{P}}_{out}$ values extracted at 25 $^\circ$C and 60 $^\circ$C. This allows to reach average temperature sensitivities ($S_{avg}$=$\Delta$ ${\hat{P}}_{out}$ $/ \Delta T$, being $\Delta T$ the size of the explored temperature range) as high as 1.4 dB/$^\circ$C. Such $S_{avg}$ value is 20 times higher than what is attainable (0.07 dB/$^\circ$C) when the thermistor included in the built SubHT is used as a separate sensor, altering the power flow between the two electrical ports of a dedicated optimized circuit exposed to the same temperature changes (Fig. 5). In addition, the SubHT shows a maximum value ($S_{max}$) for the temperature sensitivity across the investigated temperature range, defined as the magnitude of the largest slope of the ${\hat{P}}_{out}$ versus *T* trend, of 6.2dB/$^\circ$C, measured at $P_{in}$ equal to $-$ 17 dBm and around a *T* value of 57.5 $^\circ$C. In particular, we found that, by operating at such optimal working condition, the built SubHT not only exhibits the highest sensitivity but also attains the lowest temperature resolution, equal to 0.002$^\circ$C (see Supplementary Fig. 5 for a measured trend of resolution versus $P_{in}$ at 57.5 $^\circ$C). This proves that the predominant noise source limiting the value of the minimum detectable temperature change is not the adopted thermistor but the network analyzer used for the read-out. Furthermore, the SubHT shows a large dynamic range of 48 dB. The measured $S_{max}$ and dynamic range values are respectively 37 times and 35,000 times higher than the corresponding values (0.17 dB/$^\circ$C and 2.6 dB) attained when the thermistor in the SubHT circuit is used as a separate temperature sensor (Fig. 5). Finally, the adjusted R-squared^29^ value relative to the SubHT measured ${\hat{P}}_{out}$ versus *T* trend can reach 0.9669 , demonstrating a good linearity between temperature and ${\hat{P}}_{out}$. So, our measured results demonstrate that SubHTs can surpass the fundamental limits of the sensitive component in their stabilization networks. More details about the wired experimental set-up and the followed simulation approach are discussed in the Supplementary Material (Supplementary Fig. 2).

**Table 1:** Comparison with other temperature sensors. The maximum temperature sensitivity, the resolution, the power consumption and the size of the SubHT and of other previously reported counterparts, operating over different temperature ranges and relying on different sensing technologies.

Sensor prototypes	Sensing technology	Temperature range ($^\circ$C)	Max sensitivity (dB/$^\circ$C)	Resolution ($^\circ$C)	Power consumption (dBm)	Size ($\hbox {m}^2$)
This work	Parametric/off-chip	25–60	** 6.2**	**0.002**	$-$ 18.5	$43 \times 10^{-5}$
Ref.^30^	Optical/on-chip	22–27	0.058	–	8	0.21 $\times 10^{-6}$
Ref.^31^	Optical/on-chip	25–65	0.42	0.1	− 10	0.6 $\times 10^{-5}$
Ref.^32^	Optical/on-chip	26–100	0.23	–	–	63 $\times 10^{-6}$
Ref.^33^	Optical/off-chip	47–63	2.26	–	–	–
Ref.^34^	Optical/off-chip	22–40	2.1	0.0005	16.2	–
Ref.^35^	Optical/off-chip	40–100	1.65	–	–	–
Ref.^36^	Optical/off-chip	15–60	0.22	–	32	–
Ref.^37^	Optical/on-chip	30–80	0.1	0.0098	-	-
Ref.^38^	Optical/off-chip	22–60	0.13	–	–	-
Ref.^21^	SAW	25–300	0.16	–	–	–
Ref.^39^	SAW	35–118	0.13	–	–	1.2 $\times 10^{-6}$
Ref.^40^	SAW	25–300	0.065	0.15	10	7.5 $\times 10^{-6}$
Ref.^41^	SAW	20–100	–	0.016	–	3.24 $\times 10^{-6}$
Ref.^42^	CMOS	0–100	–	0.0582	-7.8	0.0137 $\times 10^{-6}$
Ref.^43^	CMOS	30–49	0.027	0.003	–	–
Ref.^44^	CMOS	− 20 to 60	–	0.21	− 69	0.15 $\times 10^{-6}$

**Figure 6:**
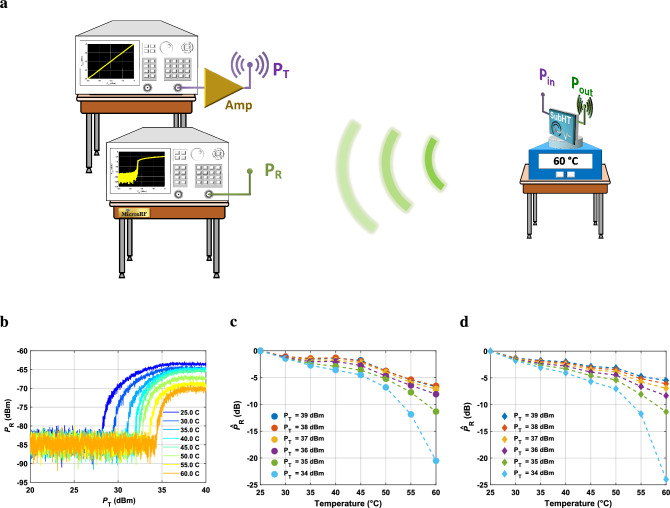
Wireless characterization of the built SubHT used as a WSN. (**a**) Overview of the wireless set-up used to characterize the SubHT as a WSN, sensing the local temperature at 4 meters away from two network analyzers placed in the RF test and characterization facility of our group (the *MicronRF* Laboratory) and together emulating a complementary interrogating node. (**b**) Measured $P_{R}$ versus $P_{T}$ trends extracted for the explored temperatures. (**c,d**) Measured (**c**) and simulated (**d**) ${\hat{P}}_{R}$versus *T* trends extracted from the wireless characterization of the built SubHT.

A comparison between the built SubHT and other recently reported intensity-level temperature sensors in terms of maximum sensitivity, resolution, power consumption and size is provided in Table 1. As evident, the measured SubHT can exhibit a higher temperature sensitivity than any other previously reported counterparts, yet not requiring any active and large sensing set-ups, such as those needed when relying on off-chip optical components and systems, or any integrated complementary metal-oxide-semiconductor (CMOS) devices, SAW devices or optical devices. In addition, due to its record-low $P_{th}$, the reported SubHT requires one of the lowest power to operate, thus providing the necessary means to achieve a large communication range when used as a WSN. Ultimately, it is worth pointing out that since SubHTs operating as temperature sensors rely on components that can be synthesized through standard CMOS processes, they can also be implemented on-chip when targeting the highest degree of miniaturization.

After characterizing its sensing capabilities through a wired set-up, we designed a new experiment to demonstrate the ability of the built SubHT to operate as a fully passive WSN, remotely sensing any temperature variations even when operating in uncontrolled electromagnetic environments like the authors’ laboratories at Northeastern University. In order to do so, two off-the-shelf 50 $\Omega$-matched dipole antennas were connected at the SubHT’s input and output ports. This rendered the SubHT simultaneously able to receive its interrogating signal wirelessly and to radiate its parametrically generated output signal. Moreover, two additional antennas, identical to those used by the SubHT, were connected to the same network analyzers from the previous wired characterization (Fig. 4c). This allowed to emulate a complementary wireless interrogating transceiver like the one conceptualized in Fig. 1a, able to radiate an interrogating signal at 886 MHz with power $P_{T}$ while simultaneously receiving a portion (i.e., $P_R$) of $P_{out}$ at 443 MHz. The two network analyzers were positioned 4 meters away from the SubHT and next to each other, as depicted in Fig. 6a. As in our former experiment, the SubHT was placed on top of a digitally controlled hotplate to set the temperature value at its now remote location. All the antennas were physically oriented to minimize any polarization losses that would lower the power received by the SubHT (i.e., $P_{in}$) and reduce $P_{R}$. The adoption of an additional amplification stage, connected between the output port of the network analyzer used for transmission and the adjacent antenna, allowed to sweep $P_{T}$ between 20 and 40 dBm, while varying *T* at the SubHT location as in our former wired experiment. More details regarding the adopted wireless set-up are provided in the Supplementary Material (Supplementary Fig. 3). The measured $P_{R}$ versus $P_{T}$ characteristic for the explored *T* values is reported in Fig. 6b. As evident, distinguishable and monotonic temperature-driven changes of the $P_{T}$ values triggering the sub-harmonic oscillation ($P_{th}^T$) in the SubHT can be observed, even when operating the SubHT as a WSN. In particular, $P_{th}^T$ values between 27 and 34 dBm were found as *T* was varied from 25 $^\circ$C to 60 $^\circ$C. Such high power levels are needed to compensate for the losses encountered during the electromagnetic propagation and for those introduced by all the adopted electrical components and connections. Since for $P_{T}$ higher than $P_{th}^T$ the measured $P_{R}$ values are 20 dB or less above the noise floor of the network analyzer used to extract them, the wireless sensing of *T* can be achieved across the entire explored temperature range for $P_{T}$ values higher than 34 dBm. The measured and simulated $P_{R}$ values (${\hat{P}}_{R}$ ) for all the investigated *T* values and normalized with respect to the corresponding $P_{R}$ values at 25 $^\circ$C  are reported in Fig. 6c and Fig. 6d respectively. As evident, a large difference ($\Delta$ ${\hat{P}}_{R}$) between the $P_{R}$ values at 25 $^\circ$C and 60 $^\circ$C was found for $P_{T}$ equal to 34dBm, resulting in an average sensitivity ($S_{avg}^w$) of 0.6 dB/$^\circ$C and in a dynamic range of 21 dB. A maximum measured sensitivity ($S_{max}^w$) of 3dB/$^\circ$C was detected for the same $P_{T}$ value.

A comparison between the SubHT wireless sensing performance and those of other recently reported chip-less and battery-less tag-based WSNs is shown in Table 2. Due to their different operational principles, their different interrogation schemes and their not homogeneous read-out parameters, the WSNs listed in Table 2 are compared through a set of key common operational metrics, including the interrogation frequency, the explored temperature range, the communication range and the equivalent isotropic radiated power (EIRP) of the interrogating node considered during their testing and characterization. As evident, the SubHT reported in this work permits to achieve the highest communication range. Even more, while our preliminary measurements already showed that the built SubHT enables longer communication ranges, up to 7 m, even longer ranges are expected in the future through further technological and design developments. For instance, as we theoretically discussed in^27^, the minimum parametric power threshold exhibited by any varactor-based parametric system is ultimately set by the load and source characteristic impedances that, for SubHTs, represent the input impedances of the adopted antennas. Hence, through the future use of custom low-impedance ($<10\,\Omega$) antenna designs, we anticipate to reduce this power threshold by more than 200 times, thereby enabling much longer communication ranges and higher sensitivities, which will be exclusively limited by the receiver’s power sensitivity and by the noise floor of the interrogating nodes. Finally, it is worth pointing out that the SubHT’s measured $S_{avg}^w$ and $S_{max}^w$ values exceed by nearly 4 and 19 times the corresponding ones just recently demonstrated by using advanced mm-wave imaging circuits that analyze the temperature sensitive echo generated by a 2 meters distant passive tag^45^.

**Table 2:** Comparison with other chip-less and battery-less tag-based WSNs for temperature sensing. The interrogation frequency, temperature range, communication range and EIRP relative to the reported SubHT are compared to those of other previously developed counterparts tested in uncontrolled (this work and Refs.^45–50^) or controlled (Ref.^51^) electromagnetic scenarios.

Sensor prototypes	Interrogation frequency (GHz)	Temperature range ($^\circ$C)	Communication range (m)	*EIRP* (dBm)
This work	0.886	25–60	4	37
Ref.^46^	29.75	24–33	2	–
Ref.^45^	24	25–55	2.4	44.3
Ref.^47^	3	22–109	1.5	30
Ref.^48^	2.4	27–53	0.2	36
Ref.^51^	3	20–54	3.4	–
Ref.^49^	4.5	− 20 to 0	0.2	27
Ref.^50^	0.9	–	1.5	36

## Conclusions

We have presented a novel class of chip-less and battery-less tag-based WSNs, named as subharmonic tags (SubHTs). We have showed that SubHTs are inherently able to surpass all the performance limitations of the existing harmonic tags. Also, they enable record-high sensitivities and dynamic ranges while being exclusively formed by off-the-shelf components assembled on printed substrates. These unprecedented characteristics were experimentally verified in a standard laboratory setting. In order to do so, we built the first Ultra-High-Frequency SubHT prototype designed to continuously monitor the temperature remotely from an interrogating node. The unique dynamics leveraged by the reported system and discussed for the first time in this article allowed to achieve large, electronically and passively boosted temperature sensitivity and dynamic range, up to 6.2dB/$^\circ$C and 48 dB. These values are respectively 37 and 35,000 times higher than what is possible when the commercial thermistor, selected as the SubHT temperature-sensitive component, is independently used as a temperature sensor for operation within the same explored temperature range. Also, due to its large sensitivity, a minimum temperature resolution of 0.002$^\circ$C was found. The maximum sensitivity achieved by the SubHT highly exceeds the ones attained by state-of-the-art counterparts relying on advanced on-chip manufacturing or on large optical components and systems.

## Supplementary information

Supplementary information 1
