# Supplementary material for: A chip-less and battery-less subharmonic tag for wireless sensing with parametrically enhanced sensitivity and dynamic range
Source: Sci Rep. 2021 Feb 12;11:3782. doi: 10.1038/s41598-021-82894-x (PMC7881208; doi:10.1038/s41598-021-82894-x)
Supplement: Supplementary file 1 — Supplementary information 1 [file 41598_2021_82894_MOESM1_ESM.pdf]

A chip-less and battery-less subharmonic tag for wireless sensing with parametrically enhanced sensitivity and dynamic range

Hussein M. E. Hussein[1*], **Matteo Rinaldi**[1], **Marvin Onabajo**[1], **and Cristian Cassella**[1]

[1]Northeastern University, Electrical and Computer Engineering Department, Boston, United States
[*]e-mail: h.hussein@northeastern.edu

Supplementary Figures

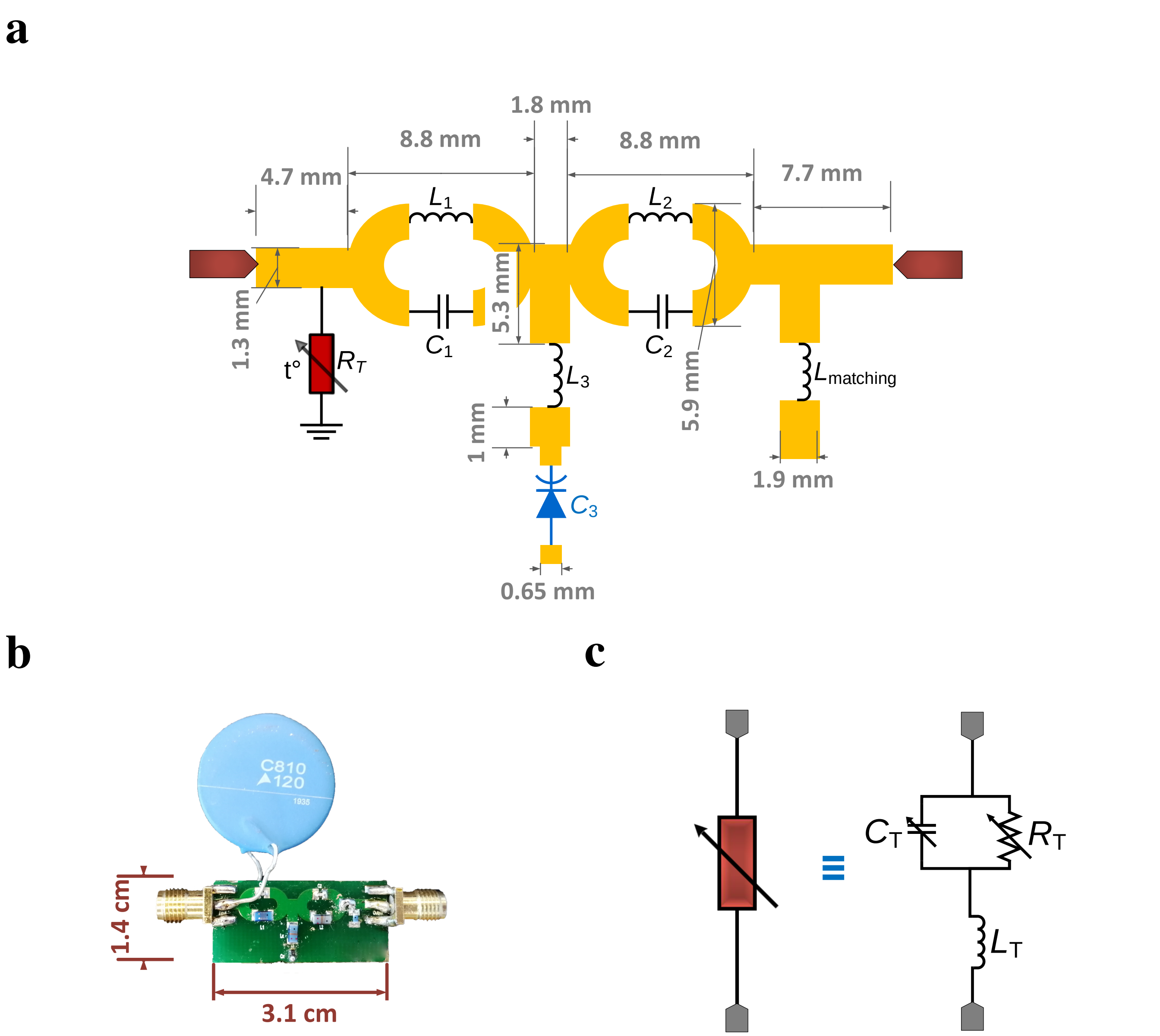

Supplementary Figure 1 | The details of the fabricated prototype of the SubHT. **a**, Layout of the PCB with the annotated connection for every component together with the dimensions of the PCB traces. **b**, A picture of the fabricated SubHT prototype assembled on a PCB of FR-4 substrate with two standard SMA connectors for the input and the output. **c**, Equivalent high frequency circuit model of the thermistor used in the SubHT prototype.

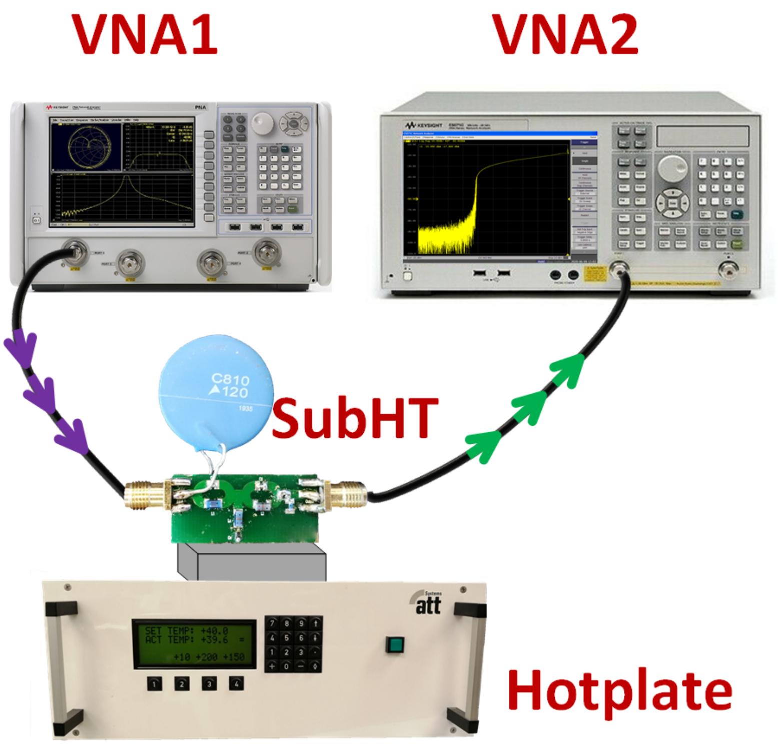

Supplementary Figure 2 | The experimental setup for the wired characterization and temperature sensing of the SubHT. During the wired characterization of the sensing characteristics of the built SubHT (see Fig. 4 in the main manuscript), we used two vector network analyzers (VNAs) and a temperature controlled hotplate. The first VNA (Keysight PNA N5221A) was continuously feeding the SubHT's input port with a signal at f_{in} = 886MHz, while sweeping the power from -25dBm to 5dBm. The second VNA (Keysight ENA E5071C) was monitoring the power of the SubHT's output port at f_{out} = 443MHz, while being triggered by a synchronization signal from the first VNA in order to collect all the measured data points. The SubHT was placed over a digitally controlled hotplate for a temperature sweep from 25°C to 60°C with steps of 2.5°C, while waiting sufficiently long for each temperature data point to make sure that a steady-state temperature at the SubHT location was reached. By relying on a commercial IR detector, we were able to determine that a waiting time of 10 minutes was sufficient for the SubHT to reach its steady-state temperature after any temperature changes during our experiment. For every temperature, we collected the power sweep data from the VNAs to monitor the temperature-driven shift of the P_{out} *vs* P_{in} characteristics.

a

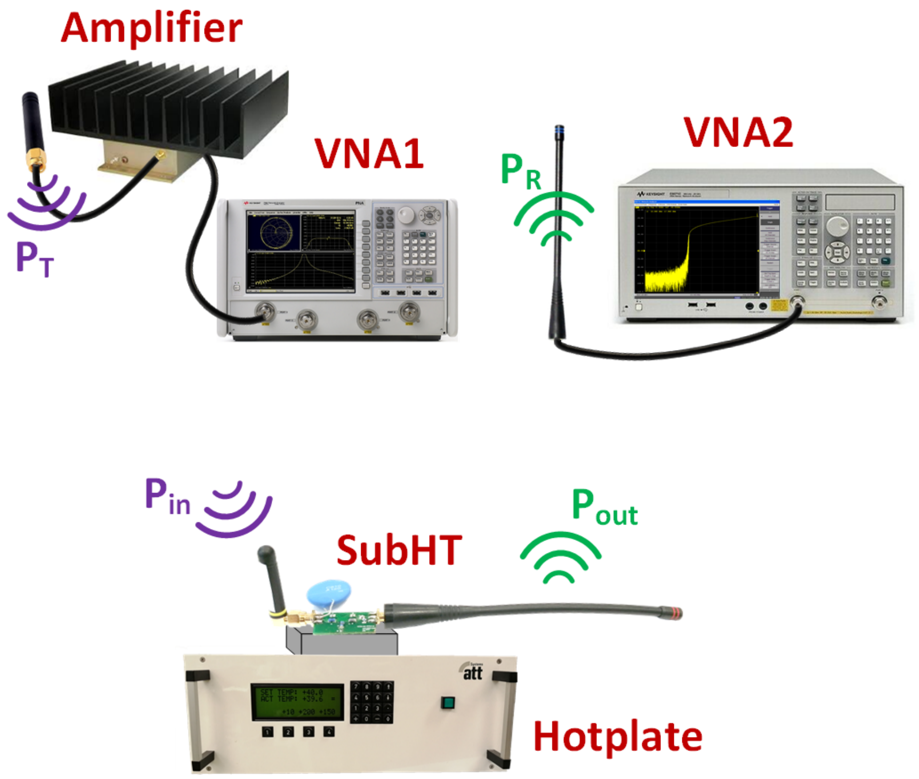

b

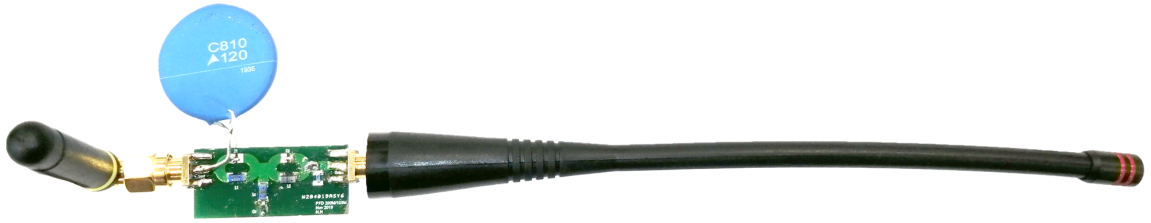

Supplementary Figure 3 | The experimental setup for the wireless temperature sensing of the SubHT. a, The experimental setup that we used during the characterization of the SubHT operation as a WSN is illustrated. As shown, the input and output ports of the SubHT were here connected to two commercial antennas. The antenna (model no. AEACAC054010-S915) connected to the SubHT input port exhibits a gain of 2dBi whereas the other antenna (model no. 712-ANT-433-CW-QW) connected to the SubHT output port exhibits a gain of 3.3dBi. An identical set of antennas was also connected to the same network analyzers that were used in our former wired setup. An amplifier (ZHL-1000-3W+, with power gain of 45dB) was used between the output port of the network analyzer operating as the transmitter and its corresponding antenna. The wireless performance of the SubHT were extracted in a laboratory space, which is an uncontrolled electromagnetic environment populated with significant levels of electromagnetic noise. Furthermore, the two VNAs were placed next to each other and 4 meters away from the built SubHT. As in our former experiment, the SubHT was positioned on the digitally controlled hotplate for temperature measurements. **b**, The SubHT prototype connected to two antennas, at the input and output ports respectively.

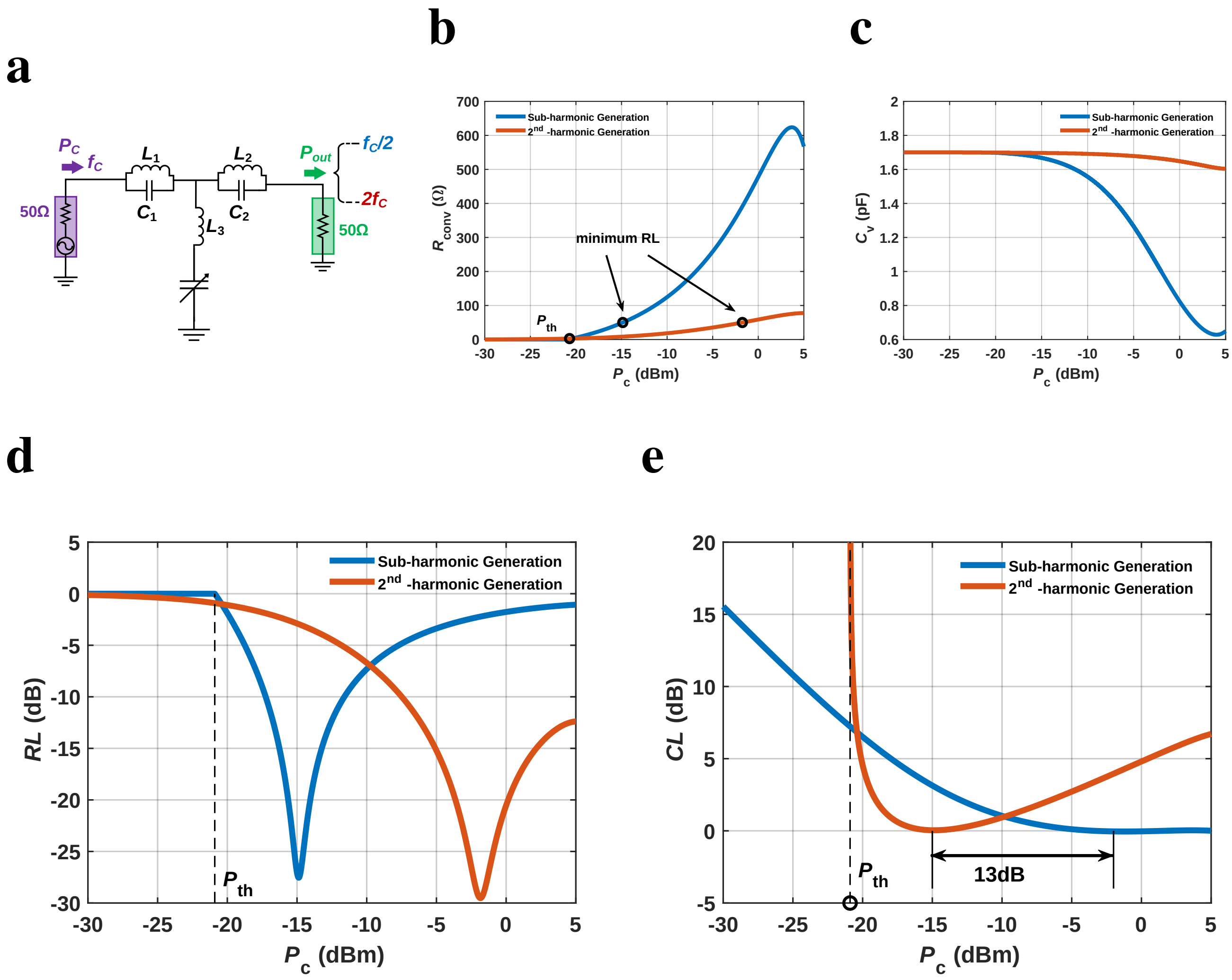

Supplementary Figure 4 | A comparison between ideal frequency doubling and frequency dividing circuits based on the same nonlinear reactance. a, Schematic of the circuit topology used for both the analyzed parametric frequency dividing circuit and the frequency doubling circuit. The values of the ideal lossless components used for both circuits are the following: dividing circuit) L_1=382.5nH, L_2=742.5nH, L_3=500nH, C_1=6.6pF, C_2=0.85pF, C_3=1.7pF; doubling circuit) L_1=28.7nH, L_2=705nH, L_3=336nH, C_1=5.5pF, C_2=0.9pF, C_3=1.7pF; **b-c**, Extracted R_{conv} (**b**) and C_v (**c**) vs. P_c for both investigated circuits; **d-e**, Extracted return-loss (RL) (**d**) and conversion loss (CL) (**e**) *vs.* P_c for both investigated circuits. All simulations were run in a commercial Harmonic Balance (HB) simulation platform set to consider an HB order in pair of 4 when considering a fundamental frequency of 100MHz.

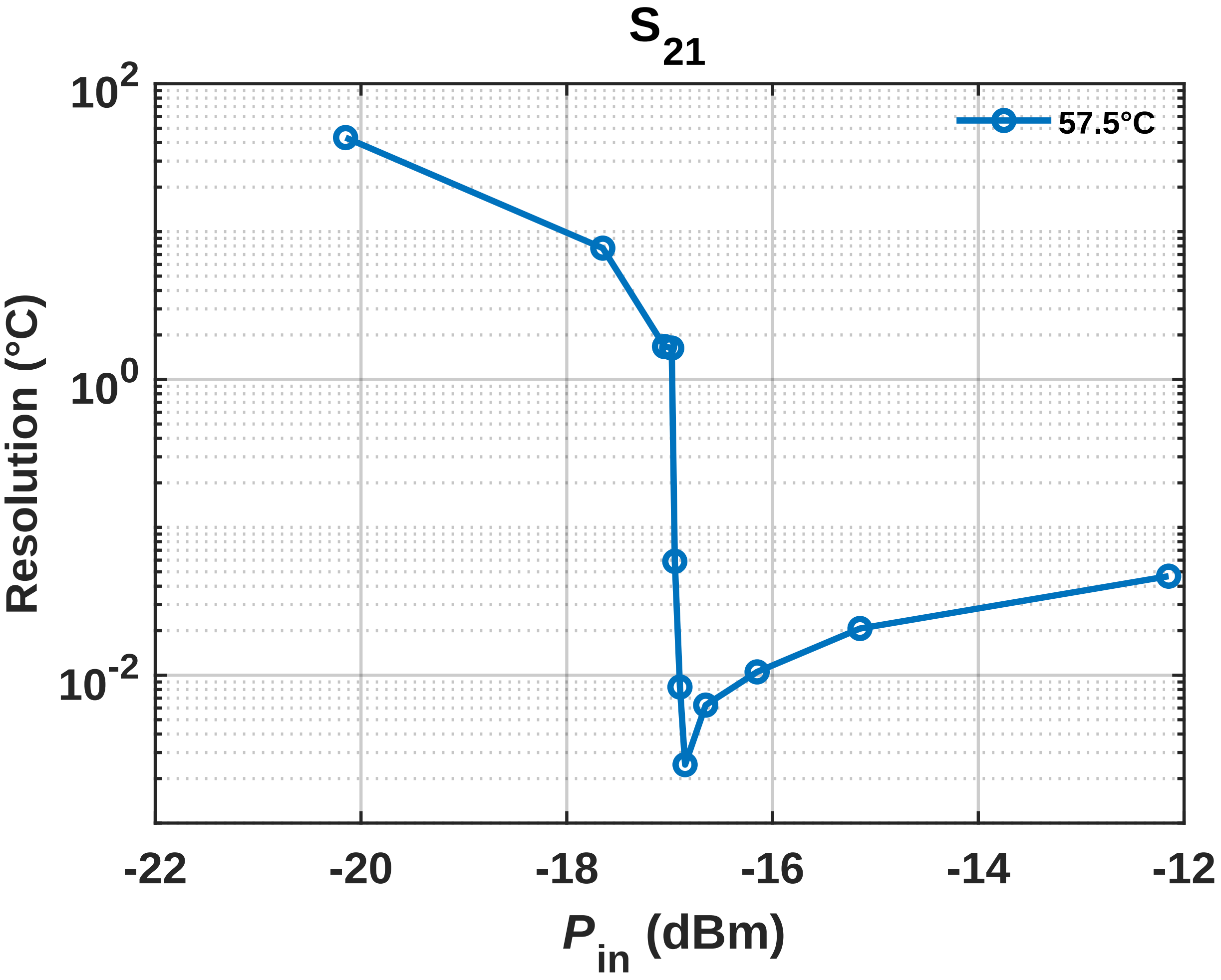

Supplementary Figure 5 | The temperature resolution of the SubHT. The plot is illustrating the temperature resolution vs. P_{in} at 57.5°C. The temperature resolution of the SubHT is calculated by obtaining the maximum noise-induced variation of P_{out} for different P_{in} values around P_{th} and divide it by the corresponding temperature sensitivity relative to the same P_{in}. It is obvious that the minimum resolution is achieved at P_{in} = -17dBm , which is the P_{in} value corresponding to the highest temperature sensitivity (S_{max}).

Supplementary Table

Supplementary Table 1. Values of the components used in the SubHT prototype.

Component	Nominal Value	Model
C_3 (varactor diode)	1.24pF	Skyworks SMV1430
C_1	1.5pF	GJM1555C1H1R5WB01
C_2	1.4pF	GJM1555C1H1R4WB01
L_1	39nH	1206CS-390XGLB
L_2	22nH	1206CS-220XGLB
L_3	22nH	1206CS-220XGLB
$L_{matching}$	1.8nH	0603HP-1N8XJLU
R_T (Thermistor)	2.6Ω	B59810C0120A070

Supplementary Notes

Supplementary Note 1. Determining Q_v in ideal lossless nonlinear circuits for frequency doubling and division

In order to determine Q_v (see Fig. 2 in the main manuscript), we designed two ideal lossless circuits characterized by the same topology and based on the same ideal nonlinear reactance, which was simply modelled in terms of a nonlinear capacitance ($C(v)$) characteristic exhibiting a tuning range of 0.3 and a chosen average capacitance (C_3). The two circuits (Supplementary Fig. 4a) were optimized to guarantee the minimum CL for low driving powers when considering the same input frequency (f_c) and the same input power (P_c) but output frequencies being twice and half f_c respectively. In other words, one circuit was designed to address a frequency doubling functionality, thus emulating the behavior of any previously reported harmonic tags, whereas the other circuit was designed to satisfy the same resonant conditions used during the design of the demonstrated SubHT[1], yet relying on the same topology used by the former frequency doubling one. Both circuits include a minimum number of components for optimum performance, equal to five, and rely on the same circuit topology. In particular, two lossless LC notch resonators (L_1-C_1 and L_2-C_2) are used at the input and output ports to prevent any energy, at the output and input frequencies respectively, from flowing towards the undesired circuit termination. Also, an additional inductor (L_3) is used by both circuits, in series to the adopted nonlinear reactance, to make sure that the input impedance relative to both the input and output ports can resonate at the corresponding frequencies (*i.e.* f_c and $f_c/2$ or $2f_c$, respectively). Finally, for the frequency doubling circuit, L_1, L_2 and L_3 are chosen, among all the possible combinations satisfying the described design constraints, to make sure that the minimum CL can be attained for the lowest P_c and when considering 50Ω terminations. The corresponding circuit components of the frequency dividing circuit are chosen, instead, to minimize P_{th}, as needed by any SubHTs. While comparing the Q_v trends vs. P_c relative to the same nonlinear reactance when used in the two described circuits would lead to the same phenomenological conclusion regardless of the chosen f_c value, a f_c value of 200MHz has been used during this analysis. This frequency has been chosen, for simplicity, since the design, operation and modelling of a corresponding parametric frequency dividing circuit, exploiting the same targeted circuit topology and input frequency, has been already discussed in full details in Ref[1]. The list of the component values adopted for both circuits in our circuit simulations is summarized in the caption of Supplementary Fig. 4. By extracting for increasing P_c values the driving voltage across the nonlinear reactance and the corresponding current flowing into the same component, we found the equivalent dynamical impedances exhibited at f_c by such nonlinear element in each circuit, in terms of both imaginary and real parts. From the imaginary parts, we found the trends of the capacitance (C_v) vs. P_c whereas, from the real parts, we extracted the trends of R_{conv} vs. P_c (see Supplementary Fig 4b and Fig 4c). Q_v was then computed, for both cases, by using Eq. 1.

$$Q_v = \frac{1}{2\pi f_c R_{conv} C_v} \tag{1}$$

Q_v allows to compare quantitatively the maximum conversion efficiency that the same nonlinear reactance can achieve when used either for frequency division or for frequency doubling, independently from the adopted terminal impedances and, consequently, from the characteristics of any possibly used matching networks. Consequently, its value is more suitable than CL to capture the difference in the conversion capability that any nonlinear reactances can exhibit when addressing a frequency conversion functionality. Yet, when searching for the minimum CL, such value is strongly dependent on the power dependent return loss (RL) at the input circuit port and, consequently, on the frequency selectivity of the adopted circuit topologies. Therefore, while the smaller Q_v exhibited by the sub-harmonic frequency

generator for P_c higher than P_{th} maps, for any given P_c, a higher R_{conv} than generated by the frequency doubling circuit, this also leads to a more sensitive trend of RL vs. P_c (see Supplementary Fig 4d) for smaller P_c values. As a result, any sub-harmonic parametric frequency generators exhibit the lowest CL (0dB, when considering all the circuit components to be lossless as we did here) for a much lower P_c value than the frequency doubling counterparts (13dB lower in this studied numerical example). Yet, they also show CL values that more rapidly increase from their optimal value as P_c is increased well beyond P_{th} (see Supplementary Fig 4e). This feature can be easily verified by noticing that the minimum CL is achieved for both investigated circuits at the same P_c values giving the minimum RL at f_c, relative to their input ports (see Supplementary Fig 4d and Fig 4e). It is worth emphasizing that the curves reported in Supplementary Fig 4 refers to the case in which all the circuit components are lossless. Much higher CL values are practically attained[2], in fact, due to the limited Q (< 80) that commercially available lumped inductors can attain.

Supplementary Note 2. Details on the SubHT design flow

In order to determine the component values of the built SubHT, we relied on the design and optimization flow that we described in ref.[1]. In particular, since we decided to use commercial antennas for this first SubHT prototype, our ultimate design goal was to minimize P_{th} when terminating the SubHT input and output ports with 50Ω, which is the input impedance of the majority of the commercial antennas available on the market. For this purpose, following our theoretical study[1] on the stability of varactor-based parametric systems and components, we selected a varactor device with a small capacitance (C_0) when left unbiased and characterized by the largest capacitance tuning range we could find. Yet, we avoided using any varactors with a C_0 values smaller than 200fF, as this would have resulted in the need of inductors that are too large to avoid self-resonance at 886MHz. By relying on our previously constructed simulation and design frameworks and by using a circuit topology that includes a shunted inductor before the output port (to reduce the electrical loading caused by the SubHT output antenna) and a commercial thermistor after the input port, we ran an optimization process to determine the optimum values of the other electrical components of the SubHT, along with the best geometrical characteristics of the layout elements (microstrips and vias) used for interconnections. Such process allows to find the proper circuit parameters that resonate and minimize the impedances seen by the adopted variable capacitor at both 886MHz and 443MHz. This optimization process was performed in a commercial harmonic-balance (HB) simulator by relying on a simulation technique that we just recently demonstrated[1] to detect the rising of sub-harmonic oscillations in largely-modulated nonlinear dynamical systems. This technique, referred to as the *power auxiliary generator* technique, was developed to detect, the existence of bifurcations in the operation of largely-driven varactor-based circuits through standard harmonic-balance algorithms, along with the steady-state operational response exhibited by such systems. The optimal components selected after completing our optimization routine are listed in Supplementary Table 1.

Supplementary Note 3. The calculation of the temperature resolution

An other important parameter for any temperature sensors is the minimum temperature resolution, which represents the smallest temperature change that can be reliably sensed and distinguished by any existing background noise generated from stochastic fluctuations in the electrical response of the adopted temperature sensitive component (the thermistor in this case) or from those originated from the electronic equipment used for read-out. Similarly to any other intensity level sensors using the output power of a system as the means to extract the temperature information, the minimum resolution can be found from the ratio between the largest magnitude of the noise-driven output power fluctuations (ΔP_{out}) and the sensitivity of the sensor. However, differently from any previously reported temperature sensors and as shown in our main manuscripts, such ratio generally depends on the selected SubHT operational point, thus varying with P_{in} and with T. As we are interested in characterizing the minimum resolution that the built SubHT can achieve, the SubHT was made to work at the operational temperature enabling the highest temperature sensitivity (i.e. 57.5°C). Also, since the sensitivity of SubHTs is generally dependant on P_{in}, we experimentally extracted ΔP_{out} at many P_{in} values. In order to measure ΔP_{out}, we injected a continuous-wave (CW) signal at 886MHz into the input port of the built SubHT, characterized by P_{in} values ranging from -20dBm to -12dBm. On the other hand we monitored the output of the SubHT at 443MHz after setting the IF bandwidth of our measurements to 100Hz. By monitoring the time fluctuations of P_{out} for any explored P_{in} values, we were able to extract ΔP_{out} by calculating the maximum variation of P_{out} over the considered time period. Then, by using Eq. 2 and after extracting the temperature sensitivity relative to each investigated P_{in} from Fig. 4 of the main manuscript, we were able to find the trend of the resolution vs. P_{in} that, as expected, showed the minimum resolution at the P_{in} value giving the highest temperature sensitivity (Supplementary Fig. 5).

$$Resolution = \frac{\Delta P_{out}}{Sensitivity} \tag{2}$$

References

1. Hussein, H. M. E. *et al.* Systematic synthesis and design of ultralow threshold 2:1 parametric frequency dividers. *IEEE Transactions on Microw. Theory Tech.* 1–1 (2020).
2. Lazaro, A., Villarino, R. & Girbau, D. A passive harmonic tag for humidity sensing. *Int. J. Antennas Propag.* (2014).
